# Measurement properties of self-report pedestrians’ road crossing behavior questionnaires constructed based on the theory of planned behavior: protocol for a systematic review

**DOI:** 10.1186/s13643-019-1121-6

**Published:** 2019-08-03

**Authors:** Mahdi Moshki, Abdoljavad Khajavi, Fatemeh Sadeghi-Ghyassi, Homayoun Sadeghi-Bazargani, Saeid Pour-Doulati

**Affiliations:** 1 Health Education and Health Promotion Department, School of Health; Social Development & Health Promotion Research Center, Gonabad University of Medical Sciences, Gonabad, Iran; 2 Community Medicine Department, School of Medicine, Gonabad University of Medical Sciences, Gonabad, Iran; 3 Research Center for Evidence Based Medicine, Tabriz University of Medical Sciences, Tabriz, Iran; 4 Road Traffic Injury Research Center, Tabriz University of Medical Sciences, Tabriz, Iran; 5 Social Development & Health Promotion Research Center, Gonabad University of Medical Sciences, Gonabad, Iran; 6 Center for Non-Communicable Diseases Control and Prevention, East Azerbaijan Province Health Center, Tabriz, 5143814998 Iran

**Keywords:** COSMIN, Pedestrian, Questionnaire, Theory of planned behavior, Quality assessment

## Abstract

**Background:**

Pedestrians’ unsafe crossing behavior exposes them at risk of trauma and death and puts a tremendous burden on the health care system. The theory of planned behavior (TPB) is one of the leading theoretical models used to develop pedestrians**’** road crossing behavior questionnaires, yet the quality of measurement properties of them has not been evaluated. The aim of the proposed systematic review is to evaluate the quality of measurement properties of the questionnaires constructed based on the TPB to predict pedestrians**’** road crossing behavior.

**Methods:**

We will include studies validating or evaluating one or more psychometric properties of the self-reported questionnaire employing the TPB for predicting pedestrians**’** road crossing behavior. A comprehensive search strategy will be formulated based on the components of review aim. The databases of MEDLINE, Embase, PubMed, Cochrane Library, PsycINFO, PsycARTICLES, and ProQuest, also grey literature and the reference lists of the included studies, will be searched. A hand search for the relevant journals and Google Scholar will be conducted. COnsensus-based Standards for the selection of health Measurement INstruments (COSMIN) Risk of Bias checklist will be used to evaluate the measurement properties of the included questionnaires. First, we will assess standards for the methodological quality of each study. Then**,** each scale or subscale of a questionnaire will be rated using the updated criteria for good measurement property. We will quantitatively pool or qualitatively summarize the results and will evaluate them against the criteria for good measurement properties. Finally, we will grade the pooled or summarized evidence using the GRADE (Grading of Recommendations, Assessment, Development, and Evaluation) approach and provide recommendations for the most appropriate instrument.

**Discussion:**

The proposed systematic review will evaluate the measurement properties of self-report pedestrians**’** road crossing behavior questionnaires constructed based on the TPB. The findings will help researchers in selecting the appropriate TPB**-**based instrument for pedestrians’ road crossing behavior.

**Systematic review registration:**

PROSPERO CRD42017047793

**Electronic supplementary material:**

The online version of this article (10.1186/s13643-019-1121-6) contains supplementary material, which is available to authorized users.

## Background

### Rationale

Pedestrian injuries and deaths should be considered as a growing public health problem [1–3]. Pedestrians are amongst the most vulnerable road users globally, accounting for 23% of the world’s road accident deaths in 2018 [4]. Unsafe road crossing behavior exposes them to risk of trauma and death and imposes a heavy burden on the health care system [5, 6]. The United Nations (UN) recommends governments to pay more attention to vulnerable road users such as pedestrians, by making proper policies and practices for pedestrian safety [7]. The high rate of pedestrian injury and mortality highlights the importance of an urgent call for evidence-based health education and safety promotion programs [8]. Nevertheless, research and interventions to improve pedestrian safety are still unsatisfactory [4, 9, 10].

Ensuring appropriate targets for intervention [9], the theory-driven interventions are more effective than interventions lacking theory [10–12]. The theory of planned behavior (TPB) developed by Ajzen [13] is one of the most frequently applied social-psychological models and helps health professionals to identify key beliefs to develop appropriate health behavior change intervention [11, 14, 15]. A number of researchers have used the TPB to predict pedestrians’ intention to cross the road in a potentially hazardous situation [16–24]. The TPB renders an authoritative framework for conceptualization, measurement, and identifying issues affecting behavior [11]. The TPB argues that behavioral intention and perceived behavioral control (PBC) are proximal predictors of actual behavior and is determined by its three basic constructs, including attitude toward the behavior, subjective norms, and PBC [14, 22]. In order to measure intention, it is necessary to measure its predictors. These predictors are latent variables and should be measured indirectly through a questionnaire response [14].

Development of a valid and reliable questionnaire as a measurement instrument is a very critical point, particularly in social-psychological and health-related behavior research. Due to some confusing issue and diverse views about the operationalization of the TPB, particularly for non-psychologist researchers[14], Ajzen [25], Francis et al. [26], Fishbein and Ajzen [27], Conner and Norman [28], and Godin and Kok [29] proposed some sort of guidelines for constructing the TPB-based questionnaire to help the researchers in developing a valid and reliable tool. In developing a TPB-based questionnaire, initial qualitative study, the wording of questions, response formats, and scoring should be considered as essential steps [14]. Different questionnaires based on the TPB have been used to measure pedestrians’ road crossing intention/behavior worldwide [16–24, 30]. To what extent these questionnaires enjoy satisfactory measurement properties (MPs) is unknown. COSMIN (COnsensus-based Standards for the selection of health Measurement Instruments) initiative, is an international multidisciplinary team of researchers which has provided a new updated modular tool for conducting a systematic review of patient-reported outcome measures (PROMs) and evaluating the outcome measurement instruments [31]. COSMIN methodology uses the word patient but, the target population could be not the patient. In this study, the target population is pedestrian, and the outcome measure is the pedestrians’ road crossing behavior. So, here the capital letter P in the PROMs represents pedestrian instead of patient or in general let us say, participant. Systematic reviews of PROMs provide an overall understanding of the MPs of PROMs to select the most suitable PROM for a certain objective [31]. An initial search conducted within the Cochrane Database of Systematic Reviews, PubMed\MEDLINE, and PROSPERO indicates that the only systematic review for evaluating the quality of TPB-based questionnaires and their development processes has been conducted by Oluka et al. in 2014, regardless of concentration on a specific subject like pedestrian behavior [32]. Since there are no current or underway systematic reviews on the topic, a systematic review is needed and reasonable to identify, appraise, summarize, and compare the quality of MPs of available self-reported TPB-based questionnaires predicting pedestrians’ road crossing intention or behavior.

So the aim of the proposed systematic review is to evaluate the quality of MPs of the questionnaires constructed based on the theory of planned behavior (TPB) to predict pedestrians’ road crossing intention/behavior by using the updated COSMIN methodology. The following specific objectives will be achieved by the proposed systematic review.

1. To identify the existing TPB-based questionnaires predicting pedestrians’ road crossing intention or behavior
2. To evaluate the methodological quality of the included studies
3. To assess the quality of MPs
4. To grade the overall quality of evidence
5. To provide recommendation on the most suitable questionnaires

## Methods

A multidisciplinary research team which consists of the following members prepared this protocol and will conduct the main review: MM, a full professor in Health Education & Promotion, is an expert in the theory of planned behavior. SP, a Ph.D. candidate of Health Promotion, concentrated in pedestrian injury prevention and safety promotion. AK, an associate professor of Health Service Management, is an expert in systematic review methodology and measurement instruments. HS is an associate professor of Epidemiology specialist in the field of Statistical Modeling concentrated in Injury Prevention and Safety Promotion. He is also a lecturer of the JBI (Joanna Briggs Institute) Database of Systematic Reviews and Implementation Reports. FS, a Ph.D. candidate of Health Information Management, is a skilled librarian, specialist in systematic search, a member of National Research Center for Evidence-Based Medicine, and also a member of JBI and lecturer of systematic search strategies and systematic review.

This protocol has been registered in the International Prospective Register of Systematic Review (PROSPERO): CRD42017047793. We followed recommendations of the Preferred Reporting Items for Systematic Reviews and Meta-Analyses Protocols (PRISMA-P) statement [33] and the COSMIN for conducting a systematic review of PROMs [34]. The first version of COSMIN [35] only provided “standards” that concern study design and statistical methods used to assess the methodological quality of the MPs. We will use the last version of COSMIN methodology [34] which also developed “criteria” that concerns the quality of PROM and provides gold standards for good MPs. The COSMIN methodology was originally developed to use for the PROMs which are used for evaluative purposes, but it is applicable for predictive application, as suggested by its developers [30]. A PRISMA-P checklist for this protocol is available in Additional file 1.

### Inclusion criteria

The following four key elements of a systematic review of PROMs will be used to formulate eligibility criteria:

#### Construct

The construct of the study is pedestrian road crossing behavior. By road crossing behavior, we mean both safe and unsafe road crossing behavior, but not railroad crossing behavior. The first one keeps them safe from collision with road vehicles, and the second one has the potential of collision with road vehicles and subsequently could lead to injury and even death.

#### Population of interest

We will consider studies that have included pedestrians of any age.

#### Type of instrument

We will consider primary studies that have presented pedestrian self-reported TPB-based questionnaire to predict road crossing behavior or intention.

#### Measurement properties

We will consider studies that have reported on MPs concerning the content validity including face validity, structural validity, consistency, cross-cultural validity or measurement invariance, reliability, measurement error, hypothesis testing, and responsiveness.

### Inclusion criteria

We will include original studies that have employed the TPB as a theoretical framework for predicting pedestrians’ road crossing intention or behavior, where the study is a validation study or evaluating one or more psychometric properties of the self-reported questionnaire. We will not consider any restrictions on the type of population or participants, geographical location, language, setting, and time. Although we will not restrict the search strategy to the type of language, we will include only articles written in English. The non-English studies will be reported merely to inform readers about the existence of such questionnaires [31].

### Exclusion criteria

The exclusion criteria will be as follows: (1) full-text not available, (2) duplicate publications or sub-studies of included research, and (3) studies that only use self-reported TPB-based questionnaire as an outcome measurement instrument.

### Search strategy

Pertinent keywords obtained from the preliminary search were tested and verified by a qualified reviewer (FS). The agreed keywords based on the main components of the review aim and consistent with the inclusion criteria will be used to formulate the search strategy which consist**s** of a comprehensive set of search terms including index terms and free text words for:

1. *Construct of interest*: road crossing behavior
2. *Population of interest*: pedestrians
3. *Measurement properties*: content validity including face validity, structural validity, consistency, cross-cultural validity or measurement invariance, reliability, measurement error, hypothesis testing, and responsiveness
4. *Type of instrument:* self-reported TPB-based questionnaire

The Ovid MEDLINE® search strategy will be tailored to other databases. Two librarians (FS and FA) will help to formulate search strategy and then do search independently. The PRESS checklist for MEDLINE and Embase databases will be filled up by a medical librarian (Z F), who is an expert in a systematic review, and at the same time, she does not cooperate with the team. A draft search strategy for Ovid MEDLINE® is provided in Table 1.

**Table 1:** Search strategy developed for Ovid MEDLINE®

Set	Strategy
1	(behavior$ or behaviour$).tw.
2	(intention$ or predict$ or impress$ or feeling$ or attitude$ or decision$).tw.
3	(belief or believes).tw.
4	exp Behavior/ (1670182)
5	exp Intention/
6	exp Attitude/
7	1 or 2 or 3 or 4 or 5 or 6
8	cross$.tw.
9	7 or 8
10	pedestrian$.tw.
11	traffic$.tw.
12	exp Accidents, Traffic/ or exp Pedestrians/
13	10 or 11 or 12
14	9 and 13
15	exp “Surveys and Questionnaires”/
16	(questionnaire$ or inventor$ or scale$ or instrument$ or assessment$ or measure$ or survey$).tw.
17	15 or 16
18	(psychometr$ or valid$ or reliab$ or consistenc$).tw.
19	(hypothesis adj2 test$).tw.
20	(test adj2 retest).tw.
21	exp Psychometrics/
22	exp Validation Studies/
23	18 or 19 or 20 or 21 or 22
24	17 and 23
25	14 and 24
26	(reasoned adj3 action adj3 approach$).tw.
27	(theory adj5 planned adj3 behavio?r$).tw.
28	(theory adj5 reasoned adj3 action).tw.
29	(TPB or TRA or RAA).tw.
30	26 or 27 or 28 or 29
31	25 and 30
32	PEROB.tw.
33	(Pedestrian$ adj3 Road$ adj3 Cross$ adj3 Behavio?r$).af.
34	32 or 33 (13)
35	31 or 34 (20)

### Information sources

We will conduct an electronic search through the following bibliographic databases: MEDLINE (via Ovid), Embase, PubMed, Cochrane Library, PsycINFO (via EBSCO), PsycARTICLES (via EBSCO), and ProQuest. We will also search grey literature and will check the reference lists of the included studies. The databases of SCOPUS and Web of Science will be used for citation tracking to include the articles which have cited the included articles if they meet the inclusion criteria. Along with electronic search, we will fulfill a hand search for the relevant journals like Transportation Research Part F: Traffic Psychology and Behavior, and Accident analysis; and Google Scholar.

### Study records

#### Data management

We will export the results of database searches to EndNote reference management software (VX6) to manage records during the review and remove duplicate references.

#### Selection process

Two independent reviewers (SP, MM) will screen the title and then abstracts of the retrieved articles. If they meet the inclusion criteria, reviewers will read the full texts and assess them against the inclusion criteria. They will scan the reference lists of included studies to ensure literature saturation. In the case of any disagreement, the reviewer will discuss for clarification and resolution. If they could not reach a consensus, a third party (AK) will arbitrate. We will calculate inter-rater-agreement using Cohen’s *κ* to verify the level of agreement between two reviewers. Justification for the excluded articles will be provided. A PRISMA flow diagram will demonstrate included and excluded studies as depicted in Additional file 2 [36].

### Assessment of methodological quality

The COSMIN Risk of Bias (RoB) checklist will be used by two independent reviewers (SP, MM) to evaluate the MPs of the included PROMs. The COSMIN RoB checklist contains ten boxes to assess required standards for good methodological quality of single studies included in systematic reviews in terms of design requirements and statistical methods/analysis [35, 37]. We will first determine which MPs are assessed in each article to complete the corresponding box of COSMIN RoB checklist for that MP. Content validity (CV) is the most important MP because it makes clear if the items of the PROM are relevant, comprehensive, and comprehensible regarding the construct of interest and study population. So, as a general procedure, we will first evaluate PROM development and CV (boxes 1 and 2, respectively) [37]. If the PROM has sufficient CV, in the second step, we will assess the internal structure of the PROMs, including first, structural validity, followed by internal consistency, and cross-cultural validity or measurement invariance (boxes 3, 4, and 5 respectively) [37]. Otherwise, we will exclude it from the review. This step will be done if the PROM is based on a reflective model (i.e., a scale or subscale manifests itself in the items of one underlying construct). Two types of measures can be applied in the TPB model to predict intention and direct and indirect measures. The indirect measures are formative and the direct measures are reflective indicators of attitude, subjective norm, and perceived behavioral control respectively. Some researchers use only direct measures and some use both. Lastly, we will evaluate the remaining MPs including reliability, measurement error, hypotheses testing for construct validity, and responsiveness (boxes 6–7 and 9–10 respectively) [37]. Criterion validity (box 8) [37] will not be assessed because there is no gold standard for predicting pedestrians’ road crossing behavior. As such, we will not use part a of box 10 for assessing responsiveness which is based on criterion approach. We will use parts b, c, and d of the box 10 for assessing responsiveness which based on the construct approach assesses hypotheses testing by comparison with other outcome measurement instruments, between subgroups, and before and after the intervention if it is the case [34].

**Fig. 1:**
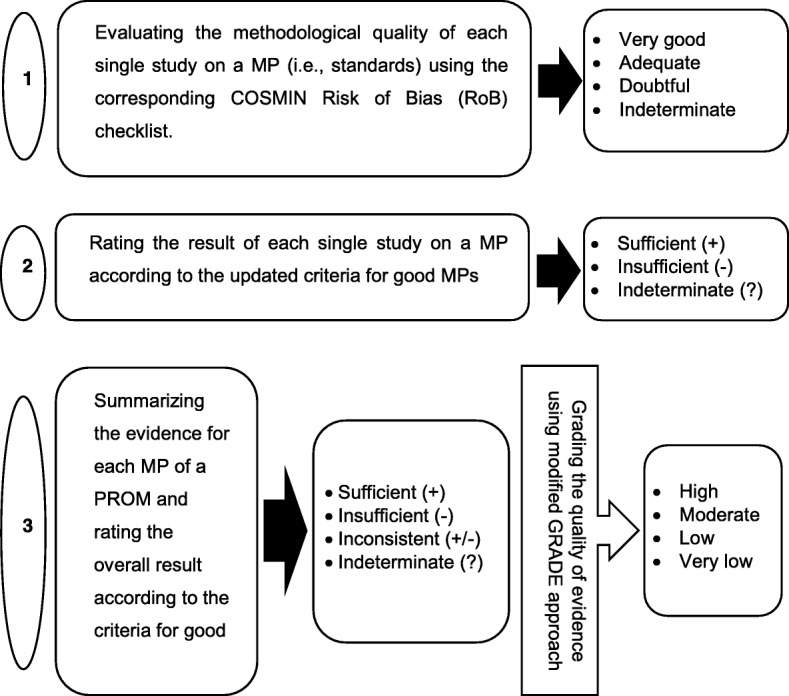
Evaluation process of each MP. Adapted from “COSMIN methodology for systematic reviews of Patient-Reported Outcome Measures (PROMs) user manual Version 1.0 dated February 2018”, by Mokkink LB, Prinsen C, Patrick DL, Alonso J, Bouter LM, de Vet HC, et al., (2018, February). Retrieved from https://www.cosmin.nl/

For the evaluation of each MP of the TPB-based pedestrian behavior, first, we will assess standards for the methodological quality of each study on MP using the corresponding COSMIN RoB checklist and applying a 4-point rating scale: very good, adequate, doubtful, and inadequate. The worst score count principal (i.e., taking the lowest score) will be employed to achieve the overall rating of the methodological quality of every single study on MP. Then, each scale or subscale of a PROM will be rated using the updated (gold) criteria for good MP as sufficient (+), insufficient (−), or indeterminate (?) [34] (Fig. 1).

### Data extraction

We will extract the data on characteristics of the PROM(s), on characteristics of the included sample(s), on results of the MPs, and on interpretability and feasibility. This information will be presented in overview tables [34]. The characteristics of the included PROMs will consist of the name of the PROMs, reference to the PROM development article, constructs being measured, language and study population for which the PROM was developed, intended context of use, available language version of the PROM, number of scales or subscales, number of items, response options, and recall period. The characteristics of the populations will consist of the geographical location, language, target population, sample size, age, gender, and setting [31]. We will contact the authors of included studies if we need any further information for clarification or obtaining missing data.

### Data synthesis

Using information taken by data extraction tables, if the results of different studies for a given MP are consistent, we will quantitatively pool, otherwise, qualitatively summarize them. The pooled or summarized results of the MP will be reported in Summary of Finding (SoF) tables [34] and will again be evaluated against the gold criteria for good MPs to get an overall rating as sufficient (+), insufficient (–), inconsistent (±), or indeterminate (?). Unlike the evaluation of the MP studies, the focus here is on the PROM itself, not on the single study. To be confident how the overall ratings are trustworthy, we will apply a modified GRADE (Grading of Recommendations, Assessment, Development, and Evaluation) approach for grading the pooled or summarized evidence as high, moderate, low, or very low (Fig. 1). Using the modified GRADE approach, we will consider four factors: risk of bias, inconsistency, imprecision, and indirectness to downgrade the quality of evidence ranging from one to three levels when the trustworthiness of the results is problematic [27]. Risk of bias refers to the methodological quality of the studies, inconsistency concerns the unexplained discrepancy of the results between studies, imprecision refers to low sample size, and indirectness means that the populations, interventions, or outcomes of the evidence differ from what the review interested in [37]. Imprecision, due to not being a matter of concern for CV, will not be considered for grading the quality of CV. COSMIN RoB checklists and detailed instructions on rating each standard are described elsewhere [38].

### Developing recommendations to use

Finally, we will classify the included questionnaires into one of the three groups:

Group “A” recommended for use and results of them would be trusted (i.e., questionnaires with sufficient content validity evidence and at least low evidence for sufficient internal consistency). Group “B” has the potential to recommend, but additional research is needed to reassess the quality of them (i.e., the questionnaires classified not in group “A” or group “C”). Group “C” will not recommend for use because there is strong evidence for an insufficient MP [31].

## Discussion

The proposed systematic review will identify and evaluate self-report questionnaires that expected to predict pedestrians’ road crossing behavior. Such a review has not been done before. The findings will reveal the existing self-report pedestrians’ road crossing behavior questionnaires constructed based on the TPB and will show the overall quality of evidence and provide recommendation for researchers to identify and select the most appropriate instrument to predict pedestrians’ road crossing intention/behavior and to determine their underlying beliefs of attitude, subjective norms, and perceived behavioral control.

## Additional files

Additional file 1: PRISMA-P 2015 checklist. (DOCX 26 kb)

Additional file 2: PRISMA flow diagram example. (DOCX 29 kb)

## Data Availability

Not applicable (**t**his is a systematic review protocol so there are no primary data available).

## Abbreviations

COSMIN: COnsensus-based Standards for the selection of health Measurement Instruments
EBSCO: Elton B. Stephens Co
Embase: Excerpta Medica database
GRADE: Grading of Recommendations, Assessment, Development, and Evaluation
MEDLINE: Medical Literature Analysis and Retrieval System Online
MP: Measurement property
PBC: Perceived behavioral control
PROSPERO: International Prospective Register of Systematic Reviews
PRISMA-P: Preferred Reporting Items for Systematic Reviews and Meta-Analyses Protocols
PROM: Patient-reported outcome measures
PsychINFO: Psychological Information Database
RoB: Risk of bias
SoF: Summary of findings
TPB: Theory of planned behavior
UN: United Nations
