# Supplementary material for: Measurement properties of self-report pedestrians’ road crossing behavior questionnaires constructed based on the theory of planned behavior: protocol for a systematic review
Source: Syst Rev. 2019 Aug 3;8:192. doi: 10.1186/s13643-019-1121-6 (PMC6679428; doi:10.1186/s13643-019-1121-6)
Supplement: Supplementary file 2 — PRISMA flow diagram example. (DOCX 29 kb) [file 13643_2019_1121_MOESM2_ESM.docx]

**Additional file 2**

**PRISMA Flow Diagram**

**Screening**

**Included**

**Eligibility**

**Identification**

Records identified through database searching
(n = )

Additional records identified through other sources
(n = )

Records after duplicates removed
(n = )

Records screened
(n = )

Records excluded
(n = )

Full-text articles assessed for eligibility
(n = )

Full-text articles excluded, with reasons
(n = )

Studies included in qualitative synthesis
(n = )

Studies included in quantitative synthesis (meta-analysis)
(n = )
